# Adverse prognostic impact of the loss of STAG2 protein expression in patients with newly diagnosed localised Ewing sarcoma: A report from the Children’s Oncology Group

**DOI:** 10.1038/s41416-022-01977-2

**Published:** 2022-10-11

**Authors:** David S. Shulman, Sonja Chen, David Hall, Anwesha Nag, Aaron R. Thorner, Stephen L. Lessnick, Kimberly Stegmaier, Katherine A. Janeway, Steven G. DuBois, Mark D. Krailo, Donald A. Barkauskas, Alanna J. Church, Brian D. Crompton

**Affiliations:** 1 Dana-Farber/Boston Children’s Cancer and Blood Disorders Center, Harvard Medical School, Boston, MA USA; 2 Nationwide Children’s Hospital, Columbus, OH USA; 3 Children’s Oncology Group, Monrovia, CA USA; 4 Center for Cancer Genomics, Dana-Farber Cancer Institute, Boston, MA USA; 5 Department of Population and Public Health Sciences, Keck School of Medicine, University of Southern California, Los Angeles, CA USA; 6 Boston Children’s Hospital, Harvard Medical School, Boston, MA USA; 7 Broad Institute, Cambridge, MA USA

**Keywords:** Sarcoma, Sarcoma, Tumour biomarkers, Paediatric cancer

## Abstract

**Background:**

Ewing sarcoma (EWS) is an aggressive sarcoma with no validated molecular biomarkers. We aimed to determine the frequency of STAG2 protein loss by immunohistochemistry (IHC) and whether loss of expression is associated with outcome.

**Methods:**

We performed a retrospective cohort study of patients with EWS enrolled to Children’s Oncology Group studies. We obtained unstained slides from 235 patients and DNA for sequencing from 75 patients. STAG2 expression was tested for association with clinical features and survival was estimated using Kaplan–Meier methods with log-rank tests.

**Results:**

In total, 155 cases passed quality control for STAG2 IHC. STAG2 expression in 20/155 cases could not be categorised with the limited available tissue, leaving 135 patients with definitive STAG2 IHC. In localised and metastatic disease, STAG2 was lost in 29/108 and 6/27 cases, respectively. Among patients with IHC and sequencing, 0/17 STAG2 expressing cases had STAG2 mutations, and 2/7 cases with STAG2 loss had STAG2 mutations. Among patients with localised disease, 5-year event-free survival was 54% (95% CI 34–70%) and 75% (95% CI 63–84%) for patients with STAG2 loss vs. expression (*P* = 0.0034).

**Conclusion:**

STAG2 loss of expression is identified in a population of patients without identifiable STAG2 mutations and carries a poor prognosis.

## Introduction

Ewing sarcoma (EWS) is an aggressive sarcoma of the bone and soft tissue that is defined by classical EWS translocations and an otherwise relatively quiet genome [1–3]. Outcomes for patients with localised disease have gradually improved to 78% 5-year event-free survival (EFS), but remain poor for patients with metastatic disease [4, 5]. Clinical features beyond stage have not been prognostic to a degree sufficient to inform risk-adapted therapy, and molecular biomarkers are therefore needed to define high-risk subgroups [6, 7]. Defining subgroups to test-risk-stratified treatment approaches is a high priority for the ~70% of patients presenting with localised disease.

STAG2 is a component of the cohesin complex and is involved in DNA looping. Recent work has demonstrated that STAG2 loss-of-function alters EWSR1-FLI1 transcriptional activity and is associated with mesenchymal features, increased migration and metastatic potential [8, 9]. In EWS, the STAG2 gene is mutated in ~15–20% of patients [1–3]. In prior studies of historical cohorts, deleterious STAG2 mutations were associated with poor outcomes. In these early genomic studies, a subgroup of patients had lost STAG2 expression by immunohistochemistry (IHC), but did not have an identified STAG2 mutation by sequencing [2, 3]. STAG2 mutations were found to co-occur with *TP53* mutations at a frequency that was greater than expected by chance and carried an especially poor prognosis [1]. The prognostic impact of *TP53* mutations in the 5–10% of patients with *TP53* mutant EWS requires further study [10]. Whether STAG2 is prognostic among patients with localised Ewing sarcoma treated with standard of care vincristine/doxorubicin/cyclophosphamide alternating with ifosfamide/etoposide (VDC/IE) remains a central question in future efforts to test-risk-stratified therapy in this population.

We studied a cohort of patients with localised and metastatic Ewing sarcoma treated with VDC/IE to determine: (a) whether dysregulated STAG2 and p53 expression can be identified through IHC in Ewing sarcoma; (b) whether alterations in protein expression are associated with deleterious gene alterations; and (c) whether loss of STAG2 and p53 are associated with clinical features and outcomes.

## Methods

### Patient eligibility and sample collection

Patients were required to have a pathologic diagnosis of EWS and be enrolled to the COG clinical trial AEWS0031 or biology studies, AEWS02B1 or AEWS07B1. AEWS0031 was a Phase 3 clinical trial that compared every 2-week cycle vs. every 3-week cycle of VDC/IE [11]. For each patient, FFPE tissue from diagnosis was requested, including one hematoxylin and eosin-stained slide and two unstained slides. The whole-genome amplified (WGA) DNA, generated from frozen tumour tissue for a previously published study, was available from a partially overlapping cohort [10]. All patients signed informed consent at the time of enrolment to AEWS0031, AEWS02B1 or AEWS07B1. Separate approval for this analysis was obtained from the Dana-Farber Cancer Institute Institutional Review Board.

### STAG2 and *TP53* immunohistochemistry

**Fig. 1:**
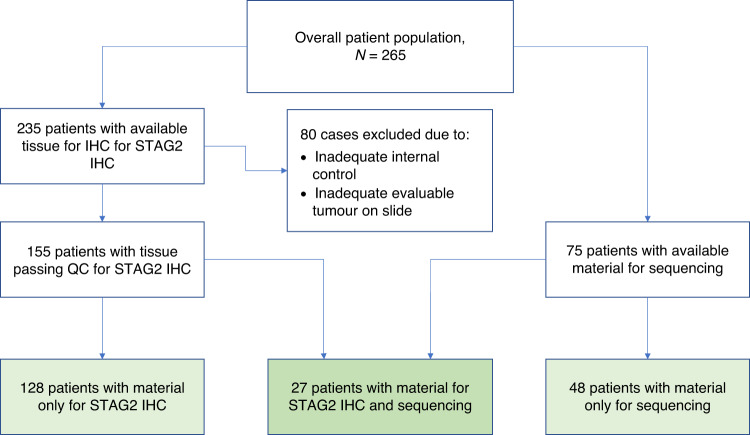
Study schema. Overview of the study schema for patients with material for STAG2 staining and for whole-genome-amplified sequencing.

Immunohistochemical staining was performed for STAG2 using the mouse anti-human monoclonal antibody (Santa Cruz SA-2 (J-12): sc-81852) and for p53 using the Leica p53-Protein antibody (DO-7). Two pathologists evaluated the percentage and intensity of nuclear staining using a semiquantitative scoring method (0 + no staining, 1+ weak staining, 2+ moderate staining, and 3+ strong staining) of stained cells without knowledge of clinical status [12, 13]. Tumours were designated to have STAG2 retained expression if >50% of cells had 2+ or 3+ staining. If >50% of tumour cells had no staining (0 + ), then these cases were considered STAG2 loss of expression, and if >50% tumour cells had 1+ intensity of staining, then these cases could not be definitively categorised as lost or expressed and were termed indeterminate (Fig. 1). No additional tissue could be requested for further analysis of the indeterminate cases. For p53, cases were designated as having absent (0–0.99% expression), normal (1–4.99%), high (5–49.99%) and ultra-high (>50%) expression. While a cut-off of 1–4.99% for normal p53 staining was arbitrarily designated, it is generally assumed that this level of expression is not likely to be associated with a pathologic process [14, 15].

Each slide was reviewed for adequacy of evaluable tissue on the slide. Cases were considered to have inadequate evaluable tumour if they had poor stain quality, a paucity of tumour on the slide, extensive tumour necrosis, or histologic features suggestive of acid decalcification such as loss of nuclear detail and nuclear/cytoplasmic eosinophilia. The presence of internal control was also required for the evaluation of STAG2 expression. Internal control was defined as positive if there was at least 1% nuclear staining of endothelial cells in associated vessels on the slide.

### STAG2 and *TP53* analysis by amplicon sequencing

Seventy-five WGA tumour DNA samples were obtained from the COG. Amplicon sequencing primers were designed to target exons of *TP53* and STAG2 (Supplemental Table 1). A total of 50 ng of WGA DNA per sample was used as starting material and processed using the TruSeq Custom Amplicon Low Input LibraryPrep (v2) kit, as per the manufacturer’s instructions (Illumina, Inc., San Diego, CA). The amplicon libraries were normalised, pooled in equimolar concentration to create a 2 nM library pool, and sequenced on one lane of the HiSeq 2500 in Rapid Run Mode.

The analysis pipeline workflow progressed as follows. Paired-end reads were aligned to the UCSC hg19 reference human genome using the Illumina banded Smith–Waterman algorithm. Read stitching was enabled, which combined the paired-end reads with an overlap of greater than 10 bases to create a single, longer read, aiding in more efficient alignment. The resulting aligned BAM files were next analysed using the Illumina Somatic Variant Caller. Variants were “passing” when given variant had (a) a variant frequency of greater than or equal to 3%, (b) a genotype quality greater than Q30, (c) no significant strand bias detected and d) did not occur in a homopolymer region.

Variant annotation was performed using the Illumina-On-Node-Annotation (IONA) tool. IONA adds annotations from dbSNP and COSMIC. The variant calls file (VCF) output from the Illumina pipeline was then annotated with Variant Effect Predictor (VEP) to identify “Best Effect” cDNA and protein changes along with HGVS “c.” (cDNA) and “p.” (protein) notation. Variants were filtered against the 6500 exome release of the Exome Sequencing Project (ESP) database. Variants represented at >1% in either the African–American or European–American populations and not in COSMIC > 2x may be considered to be germline.

### Statistical analysis

Patient characteristics were summarised descriptively. Event-free survival (EFS) was defined as the time from enrolment on a study until the event (relapse, second malignant neoplasm [SMN], or death). Patients who did not experience an EFS event were considered censored at the time of the last contact. Overall survival (OS) was defined as the time from enrolment on a study until death or until the last contact. Patients who were alive at last contact were considered censored at that time.

The predictor variables STAG2 IHC, STAG2 mutational status, and *TP53* mutational status were analysed for association with clinical features, EFS and OS. The p53 IHC staining was not thought to be associated with biologic p53 activity or *TP53* mutation status and was not analysed as a predictor variable. Each predictor variable was tested for association with clinical features using Fisher’s exact test for categorical clinical features or a pooled *t* test of means for continuous clinical features. The relationship between each of the three predictor variables and risks for EFS event and death were assessed with log-rank tests [16].

Womer et al. demonstrated that risk for EFS event among patients treated on AEWS0031 was significantly related to age at study enrolment (<18/≥18 years), site of the primary tumour (pelvis/other site) and randomised treatment assignment (standard timing/intensive timing) [11]. To assess the contribution of STAG2 loss of expression to risk of EFS event, a multivariate relative hazard regression model was fitted including STAG2 expression and the three established prognostic factors in the cohort of 107 patients from AEWS0031 [16]. The Wald test for the hypothesis of no association between STAG2 expression and risk for EFS when the other variables were considered in the model was calculated.

Cumulative incidence analysis was performed for local relapse, distant relapse, and local plus distant relapse for patients who were enrolled on AEWS0031 and stratified by STAG2 IHC. The *P* values presented for the cumulative incidence analysis are the Grey *P* values. Statistical evaluation was performed in SAS (Version 9.4). A *P* value ≤ 0.05 was considered significant.

## Results

### Patient characteristics

Among the overall patient cohort of 265 patients, 203 patients were included in at least one statistical analysis (128 patients with only material for STAG2 IHC, 27 patients with material for both STAG2 IHC and sequencing and 48 with material only for sequencing; Fig. 1). Characteristics of the 203 contributory patients were similar to the overall cohort of 265 patients (Table 1).

**Table 1:** Patient characteristics for 203 patients with material available for STAG2 IHC and/or mutational analysis compared to the full patient cohort (*n* = 265).

	Patients with available material for analysis, *n* = 203	All patients, *n* = 265
Study enrolment		
AEWS0031	172 (84.7%)	215 (81.1%)
AEWS02B1	21 (10.3%)	36 (13.6%)
AEWS07B1	10 (4.9%)	14 (5.3%)
Age category		
<10 years old	63 (30.5%)	75 (28.3%)
10–17 years old	128 (63.1%)	164 (61.9%)
18 years or older	13 (6.4%)	26 (9.8%)
Age at enrolment in years		
Mean (range)	12.4 (0.7–45.5)	12.8 (6.6–45.5)
Sex		
Male	115 (56.7%)	151 (53.0%)
Female	88 (43.3%)	114 (47.0%)
Race		
White	184 (90.6%)	235 (88.7%)
Black	3 (1.5%)	6 (2.3%)
Other	7 (3.4%)	9 (3.4%)
Unknown	9 (4.4%)	15 (5.7%)
Ethnicity		
Non-Hispanic	181 (89.2%)	237 (89.4%)
Hispanic	19 (9.4%)	24 (9.1%)
Unknown	3 (1.5%)	4 (1.5%)
Stage		
Localised	173 (85.2%)	218 (82.3%)
Metastatic	30 (14.8%)	47 (17.7%)
Primary site		
Non-pelvic	175 (86.2%)	227 (85.7%)
Pelvic	28 (13.8%)	38 (14.3%)
Chemotherapy regimen		
Standard timing	93 (45.8%)	117 (44.2%)
Intensive timing	79 (38.9%	98 (36.9%)
Unknown	31 (15.3%)	50 (18.9%)
STAG2 IHC (*n* = 155)		
Loss of expression	35 (22.6%)	
Retained expression	100 (64.5%)	
Indeterminate	20 (12.9%)	
STAG2 mutation (*n* = 75)		
Yes	9 (12.0%)	
No	66 (88.0%)	
*TP53* mutation (*n* = 75)		
66 (88.0%), yes	8 (10.7%)	
66 (88.0%), no	67 (89.3%)	

### STAG2 and p53 staining in patients with Ewing sarcoma

The characteristics of STAG2 staining are described in Table 2. Among patients with localised tumours, 79/125 (63.2%) patients had retained STAG2 staining, 29/125 (23.2%) patients had STAG2 loss of expression, and 17/125 (13.6%) patients had staining that could not be categorised (indeterminate). Within the cohort of patients with metastatic disease, 21/30 (70.0%) had retained STAG2 expression, 6/30 (20.0%) had STAG2 loss of expression, and 3/30 (10.0%) had indeterminate staining. Excluding indeterminate cases, 26.9% and 22.2% of cases had STAG2 loss of expression among patients with localised and metastatic disease, respectively.

**Table 2:** Biomarker characteristics for 203 patients with evaluable STAG2 IHC and/or whole-genome amplified material for amplicon sequencing.

		IHC cohort	Patients with both IHC and sequencing	Sequencing cohort	
**Localised cohort (*n* = 173)**		*n* = 125 (98 + 27)	*n* = 27	*n* = 75 (48 + 27)	
	**STAG2 IHC**		***STAG2*** **mutated/ total sequenced**	***STAG2*** **mutation**	
STAG2 analysis	Expressing	79 (63.2%)	0/17 (0.0%)	Mutated	9 (12.0%)
	Loss	29 (23.2%)	2/7 (28.6%)	Wild-type	66 (88.0%)
	Indeterminate	17 (13.6%)	1/3 (33.3%)		
	***TP53*** **mutation**			***TP53*** **mutation**	
TP53 analysis	Mutated		1 (3.7%)	Mutated	8 (10.7%)
	Wild-type		26 (96.3%)	Wild-type	67 (89.3%)
**Metastatic cohort (*n* = 30)**					
STAG2 analysis	Expressing	21 (70.0%)			
	Loss	6 (20.0%)			
	Indeterminate	3 (10.0%)			

p53 immunohistochemistry demonstrated variable staining in each case. Among 210 cases with evaluable staining for p53, 49 cases (23.3%) had an absent expression, 82 cases (39.0%) had normal expression, 37 cases (17.6%) had high expression and 16 cases (7.6%) had ultra-high expression. Twenty-six cases (12.4%) could not be definitively categorised. The rate of absent, high, and ultra-high p53 expression (48.6%) is much higher than the rate of loss-of-function mutations reported for *TP53* in Ewing sarcoma [1–3]. As has been previously reported, IHC staining for p53 appears to be an unreliable means of evaluation of *TP53* loss of function in the cancer cell and thus no formal statistical analysis was performed [15].

### STAG2 and *TP53* loss-of-function mutations identified in a subset of patients with localised disease

Deleterious *TP53* mutations were identified in 8/75 (10.7%) of patients with localised EWS and available DNA. These mutations were all missense variants. Nine of 75 (12.0%) patients with localised EWS had deleterious STAG2 mutations, with 6 patients having missense mutations and 3 having frameshift mutations (Table 2). In total, 4/75 (5.3%) patients had co-occurring STAG2 and *TP53* mutations.

Twenty-seven cases had both evaluable STAG2 staining and material for STAG2 sequencing. Within this cohort, 0/17 patients with STAG2 expressing tumours by IHC had deleterious STAG2 mutations. Only 2/7 patients with STAG2 loss of expression had detectable deleterious STAG2 mutations.

### Loss of STAG2 expression is associated with poor outcomes independent of clinical features

There were no significant associations found between STAG2 expression and clinical features for the 135 patients with categorizable STAG2 IHC data (Table 3), or among patients with localised disease treated on AEWS0031.

**Table 3:** Associations between STAG2 immunohistochemistry and clinical features.

	STAG2 expression, *n* = 100	STAG2 lost expression, *n* = 35	*P* value
Study enrolment			
AEWS0031	79 (79%)	28 (80.0%)	0.88
AEWS02B1	14 (14%)	4 (11.4%)	
AEWS07B1	7 (7%)	3 (8.6%)	
Age category			
<10 years old	31 (31%)	14 (40%)	0.67
10–17 years old	61 (61%)	19 (54.3%)	
18 years or older	8 (8%)	2 (5.7%)	
Age at enrolment in years			
Mean (range)	12.5 (1.2–33.1)	11.3 (0.9–21.3)	0.23
Sex			
Male	60 (60%)	19 (54.3%)	0.56
Female	40 (40%)	15 (45.7%)	
Race			
White	89 (89%)	33 (94.3%)	0.44
Black	1 (1%)	1 (2.9%)	
Other	5 (5%)		
Unknown	5 (5%)	1 (2.9%)	
Ethnicity			
Non-Hispanic	88 (88%)	31 (88.6%)	0.74
Hispanic	11 (11%)	3 (8.6%)	
Unknown	1 (1%)	1 (2.9%)	
Stage			
Localised	79 (79%)	29 (82.9%)	0.81
Metastatic	29 (29%)	6 (17.1%)	
Primary site			
Non-pelvic	83 (83%)	31 (88.6%)	0.59
Pelvic	17 (17%)	4 (11.4%)	
Chemotherapy regimen			
Standard timing	40 (40%)	15 (42.9%)	0.97
Intensive timing	39 (39%)	13 (37.1%)	
Unknown	21 (21%)	7 (20.0%)	

Analysis for the association between STAG2 and survival among patients with localised disease was conducted using data from patients with localised tumours enrolled to AEWS0031 with evaluable staining (*n* = 107). This population of patients did not differ significantly from the 75 patients from AEWS0031 with available sequencing material or the overall AEWS0031 study population (*n* = 511; Supplemental Table 2).

**Fig. 2:**
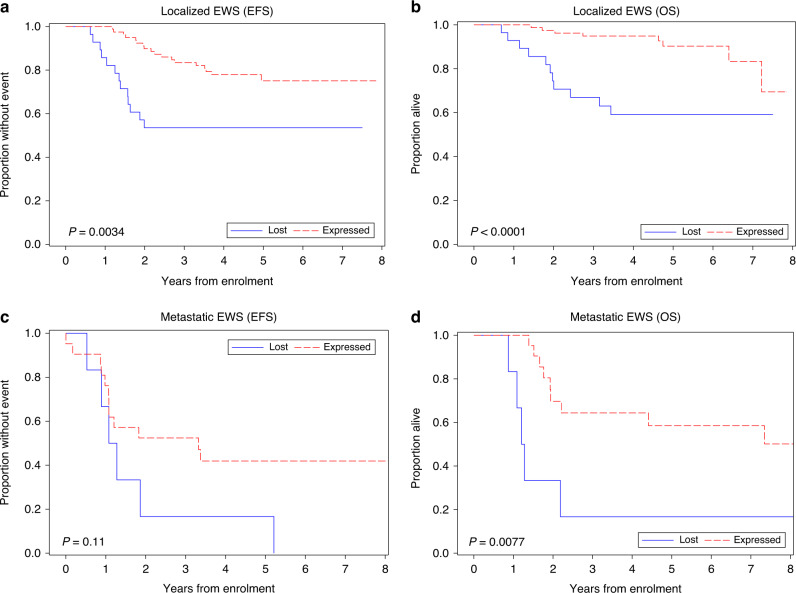
Survival stratified by STAG2 expression. Event-free survival (EFS) (**a**) and overall survival (OS) (**b**) for patients with localised Ewing sarcoma, and EFS (**c**) and OS (**d**) for patients with metastatic Ewing sarcoma stratified by STAG2 expression (STAG2 lost in blue; STAG2 expressed in red dashed).

Within this cohort, 79/107 (73.8%) had retained STAG2 expression and 28/107 (26.2%) had STAG2 loss of expression. Patients with STAG2 loss of expression had worse EFS and OS compared to patients with retained STAG2 expression (5-year EFS was 75.1% for expressed and 53.6% for lost, *P* = 0.0034; 5-year OS was 90.3% for expressed and 59.1% for lost, *P* < 0.001; Fig. 2a, b). In a multivariate analysis, STAG2 loss of expression remained associated with inferior EFS independent of other variables in the model and was the only significant variable in the model (HR = 3.00, *P* = 0.0032; Supplemental Table 3).

Among the 75 patients with available material for STAG2 amplicon sequencing, there was no difference in EFS (*P* = 0.18) or OS (*P* = 0.10) for patients with STAG2 mutated vs. STAG2 wild-type tumours (Supplemental Fig. 1A, B). There was no statistically significant association between STAG2 expression and EFS among patients with metastatic disease (*P* = 0.11); however, patients with STAG2 loss of expression had worse OS (5-year OS was 58.5% for expressed and 16.7% for lost, *P* = 0.0077; Fig. 2c, d).

**Fig. 3:**
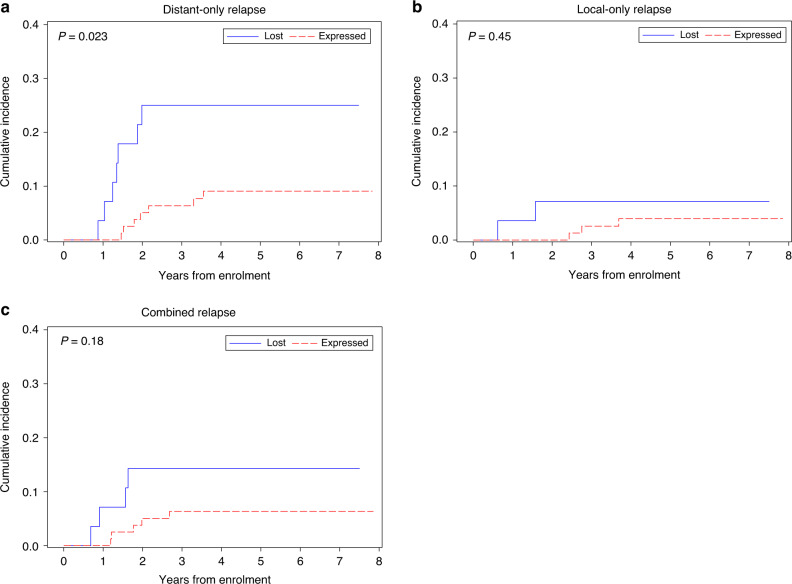
Cumulative incidence of relapse stratified by STAG2 expression. Cumulative incidence of the event by STAG2 IHC status for distant only relapse (**a**; *n* = 14), local only relapse (**b**; *n* = 5), and combined local and distant relapse (**c**; *n* = 9; STAG2 lost in blue; STAG2 expressed in red dashed).

Patients with STAG2 loss of expression had a higher incidence of metastatic relapse than patients with retained STAG2 expression (*P* = 0.023; Fig. 3a). The incidence of local and combined local and metastatic relapse did not differ by STAG2 expression status (Fig. 3b, c).

### *TP53* and TP53-STAG2 co-occurring mutations carry poor prognosis

Patients with *TP53* mutations had inferior EFS (*P* = 0.047) and OS (*P* = 0.0022; Supplemental Fig. 2A, B) compared to patients with wild-type tumours. While EFS did not differ significantly across groups defined by the presence of STAG2 and *TP53* mutation (*P* = 0.072; Supplemental Fig. 3A), patients with mutations in both *TP53* and STAG2 had the lowest 5-year OS estimate (5-year EFS was 25% for *TP53*^*mut*^*/STAG2*^*mut*^ vs. 50% or greater in the other groups; *P* = 0.0045, Supplemental Fig. 3B).

## Discussion

Loss-of-function alterations in STAG2 were associated with poor outcomes in the landmark Ewing sarcoma genomic landscape studies, and in some cases loss of STAG2 expression was identified without a known mutation [1–3]. Our study builds on this literature by identifying a population of patients with STAG2 loss of expression and no identifiable STAG2 genetic alteration. We show that among patients with localised tumours treated with VDC/IE, STAG2 loss of expression is associated with poor outcomes independent of clinical features.

This study is the first to demonstrate in a large cohort of patients with localised disease that STAG2 loss of expression was readily identifiable in a population of patients without STAG2 mutations. While prior reports suggested that STAG2 loss may occur in the absence of a gene mutation [2, 3], no prior reports have examined a large population of patients with newly diagnosed localised disease. Future studies are needed to determine the exact means through which STAG2 loss of expression occurs.

Our study is also the first to demonstrate in a large cohort of patients with localised EWS treated with VDC/IE that STAG2 loss of expression is associated with inferior EFS and OS compared to patients with retained STAG2 expression. Interestingly, although STAG2 loss of expression was not associated with metastatic stage at diagnosis, patients with STAG2 loss of expression had a higher incidence of metastatic relapse compared to patients with STAG2 retained expression. Such a biomarker is advantageous in that it can be assessed rapidly at diagnosis. Once validated in an independent cohort, STAG2 expression could be incorporated in a prospective trial studying novel treatments for patients with high-risk localised tumours. At the present time, we favour validation of this finding in a separate cohort of patients with localised Ewing sarcoma with contemporarily obtained samples before incorporating this marker into future prospective trials.

In our study, we analysed *TP53* mutation status of an overlapping cohort of patients to those analysed by Lerman et al., who did not find a significant association between deleterious *TP53* mutations and outcomes [10]. These discrepancies may be reconciled by the fact that the cohorts were relatively small, that the effect size of *TP53* mutations on outcomes may be small, and that more clinical data are now available to support a clearer determination of which variants are pathogenic or likely pathogenic. We are the first to analyse this cohort of patients with localised EWS for STAG2 and *TP53* mutations and identify worse overall survival for patients with co-occurring STAG2 and *TP53* mutations.

The overall cohort of patients with evaluable STAG2 IHC slides had slightly worse EFS when compared to contemporary cohorts of patients with EWS treated with similar regimens. One possible reason is that patients who originally presented with larger, and more aggressive tumours may have been more likely to have available tumour tissue. A limitation to our study was that we were evaluating IHC on patients who had presented often 10–15 years prior. Many samples could not be evaluated given poor slide quality or lack of internal control. We expect that far fewer patients would have invaluable IHC results using tissue that was processed on a more contemporary study and when additional slides could be requested [17]. The fact that we were able to assess STAG2 IHC with so few unstained slides that had been collected more than a decade previously speaks to the durability of STAG2 as a potential biomarker. Finally, our study is limited by the fact that our cohort did not include all clinical features and key molecular features, including *TP53* and STAG2 mutation status, for all patients. While we present data from the largest currently available cohort of patient with localised EWS treated with VDC/IE, our group is currently studying a follow-up cohort of patients treated on AEWS1031, in which we will study STAG2 expression in the context of rich clinical data, and STAG2 and *TP53* sequencing, as well as other relevant molecular biomarkers including 1q^gain^ and 16q^loss^.

In summary, we present data demonstrating that IHC identifies patients with STAG2 loss more often than sequencing and that this molecular marker identifies a subgroup of patients with high-risk localised disease. Validation of STAG2 loss in a large cohort with more comprehensive sequencing and annotated clinical features will be essential to moving towards an integral biomarker suitable for testing treatment stratification.

## Supplementary information

Nature Springer Checklist

Supplemental materials

## Data Availability

Additional data that support the findings of this study are available from the corresponding author upon reasonable request.

## References

[1] Tirode F, Surdez D, Ma X, Parker M, Le Deley MC, Bahrami A (2014). Genomic landscape of Ewing sarcoma defines an aggressive subtype with co-association of STAG2 and *TP53* mutations. Cancer Discov.

[2] Crompton BD, Stewart C, Taylor-Weiner A, Alexe G, Kurek KC, Calicchio ML (2014). The genomic landscape of pediatric Ewing sarcoma. Cancer Discov.

[3] Brohl AS, Solomon DA, Chang W, Wang J, Song Y, Sindiri S (2014). The genomic landscape of the Ewing Sarcoma family of tumors reveals recurrent STAG2 mutation. PLoS Genet.

[4] Leavey PJ, Laack NN, Krailo MD, Buxton A, Randall RL, DuBois SG (2021). Phase III Trial Adding Vincristine-Topotecan-Cyclophosphamide to the Initial Treatment of Patients With Nonmetastatic Ewing Sarcoma: A Children’s Oncology Group Report. J Clin Oncol.

[5] Grier HE, Krailo MD, Tarbell NJ, Link MP, Fryer CJ, Pritchard DJ (2003). Addition of ifosfamide and etoposide to standard chemotherapy for Ewing’s sarcoma and primitive neuroectodermal tumor of bone. N. Engl J Med.

[6] Karski EE, McIlvaine E, Segal MR, Krailo M, Grier HE, Granowetter L (2016). Identification of discrete prognostic groups in Ewing sarcoma. Pediatr Blood Cancer.

[7] Rodriguez-Galindo C, Liu T, Krasin MJ, Wu J, Billups CA, Daw NC (2007). Analysis of prognostic factors in Ewing sarcoma family of tumors: review of St. Jude Children’s Research Hospital studies. Cancer.

[8] Adane B, Alexe G, Seong BKA, Lu D, Hwang EE, Hnisz D (2021). STAG2 loss rewires oncogenic and developmental programs to promote metastasis in Ewing sarcoma. Cancer Cell.

[9] Surdez D, Zaidi S, Grossetete S, Laud-Duval K, Ferre AS, Mous L (2021). STAG2 mutations alter CTCF-anchored loop extrusion, reduce cis-regulatory interactions and EWSR1-FLI1 activity in Ewing sarcoma. Cancer Cell.

[10] Lerman DM, Monument MJ, McIlvaine E, Liu XQ, Huang D, Monovich L (2015). Tumoral *TP53* and/or CDKN2A alterations are not reliable prognostic biomarkers in patients with localized Ewing sarcoma: a report from the Children’s Oncology Group. Pediatr Blood Cancer.

[11] Womer RB, West DC, Krailo MD, Dickman PS, Pawel BR, Grier HE (2012). Randomized controlled trial of interval-compressed chemotherapy for the treatment of localized Ewing sarcoma: a report from the Children’s Oncology Group. J Clin Oncol.

[12] Fitzgibbons PL, Dillon DA, Alsabeh R, Berman MA, Hayes DF, Hicks DG (2014). Template for reporting results of biomarker testing of specimens from patients with carcinoma of the breast. Arch Pathol Lab Med.

[13] Fedchenko N, Reifenrath J (2014). Different approaches for interpretation and reporting of immunohistochemistry analysis results in the bone tissue - a review. Diagn Pathol.

[14] Yan YH, Chen SX, Cheng LY, Rodriguez AY, Tang R, Cabrera K (2021). Confirming putative variants at </= 5% allele frequency using allele enrichment and Sanger sequencing. Sci Rep..

[15] Yemelyanova A, Vang R, Kshirsagar M, Lu D, Marks MA, Shih IM, et al. Immunohistochemical staining patterns of p53 can serve as a surrogate marker for *TP53* mutations in ovarian carcinoma: an immunohistochemical and nucleotide sequencing analysis. Mod Pathol. 2011;24:1248–53. 10.1038/modpathol.2011.85 21552211

[16] Kalbfleisch J, Prentice R. The statistical analysis of failure time data, Second Edition. New York: John Wiley and Sons; 2002.

[17] Bertheau P, Cazals-Hatem D, Meignin V, de Roquancourt A, Verola O, Lesourd A (1998). Variability of immunohistochemical reactivity on stored paraffin slides. J Clin Pathol.

