# Supplementary material for: Adverse prognostic impact of the loss of STAG2 protein expression in patients with newly diagnosed localised Ewing sarcoma: A report from the Children’s Oncology Group
Source: Br J Cancer. 2022 Oct 11;127(12):2220–6. doi: 10.1038/s41416-022-01977-2 (PMC9726932; doi:10.1038/s41416-022-01977-2)

Supplemental Table 1. Regions of *STAG2* and *TP53* targeted by amplicon sequencing.

Gene	Chromosome	Start Coordinate	Stop Coordinate
TP53	17	7572930	7573008
TP53	17	7573927	7574033
TP53	17	7576535	7577160
TP53	17	7577499	7577608
TP53	17	7578172	7578559
TP53	17	7579307	7579917
STAG2	X	123156478	123156521
STAG2	X	123159690	123159768
STAG2	X	123164811	123164975
STAG2	X	123171377	123171473
STAG2	X	123176419	123176495
STAG2	X	123179014	123179218
STAG2	X	123181204	123181355
STAG2	X	123182855	123182928
STAG2	X	123184036	123184159
STAG2	X	123184966	123185249
STAG2	X	123189978	123190085
STAG2	X	123191716	123191827
STAG2	X	123195074	123195191
STAG2	X	123195621	123195724
STAG2	X	123196747	123197060
STAG2	X	123197698	123197901
STAG2	X	123199726	123199796
STAG2	X	123200020	123200291
STAG2	X	123202414	123202506
STAG2	X	123204999	123205173
STAG2	X	123210182	123210321
STAG2	X	123211807	123211908
STAG2	X	123215230	123215378
STAG2	X	123217271	123217399
STAG2	X	123220397	123220620
STAG2	X	123224420	123224819
STAG2	X	123227868	123227994
STAG2	X	123229222	123229299
STAG2	X	123234424	123234444

Supplemental Table 2. Sub-group analysis of patients with localized Ewing sarcoma treated on AEWS0031 with comparison to the entire study population.

	**Interpretable STAG2 IHC Cohort** N=107	***STAG2*** **Sequencing Cohort** N= 75	**Overall AEWS0031 Population** N=511
**Age**			
Mean (Range)	12.0 (0.9, 33.1)	12.3 (3.4, 33.1)	12.7 (0.3, 40.3)
**Age Category**			
<10 years	38 (35.5%)	23 (30.7%)	152 (29.7%)
10 to 17 years	60 (56.1%)	47 (62.7%)	302 (59.1%)
18+ years	9 (8.4%)	5 (6.7%)	57 (11.2%)
**Sex**			
Male	57 (53.3%)	41 (54.7%)	271 (53.0%)
Female	50 (46.7%)	34 (45.3%)	240 (47.0%)
**Race**			
White	96 (89.7%)	71 (94.7%)	454 (88.9%)
Black	2 (1.9%)	1 (1.3%)	13 (2.5%)
Other	4 (3.7%)	2 (2.7%)	20 (3.9%)
Unknown	5 (4.7%)	1 (1.3%)	24 (4.7%)
**Ethnicity**			
Non-Hispanic	97 (90.7%)	69 (92.0%)	459 (89.8%)
Hispanic	10 (9.3%)	6 (8.0%)	44 (8.6%)
			8 (1.6%)
**Primary site**			
Pelvic	17 (84.1%)	10 (13.3%)	452 (88.5%)
Non-pelvic	90 (15.9%)	65 (86.7%)	59 (11.5%)
**Chemotherapy regimen**	55 (51.4%)	49 (65.3%)	254 (49.7%)
Standard Timing	52 (48.6%)	26 (34.7%)	257 (50.3%)
Intensive Timing			

Supplemental Table 3. Multivariable analysis of event-free survival for AEWS0031 patients with localized Ewing sarcoma (n=107)

Variable	HR	P-Value	95% C.I. for HR
STAG2 IHC (Loss / Expressed)	3.00	0.0032	(1.44 - 6.23)
Age (≥ 18 years / < 18 years at diagnosis)	2.62	0.056	(0.98 - 7.037)
Site (Pelvic / Non-Pelvic primary tumor)	2.022	0.11	(0.86 - 4.77)
Randomized Regimen (Interval compressed / Standard)	0.81	0.58	(0.39 - 1.70)

Supplemental Figure 1. Event-free survival (A) and overall survival (B) for patients with localized Ewing sarcoma stratified by *STAG2* mutational status.

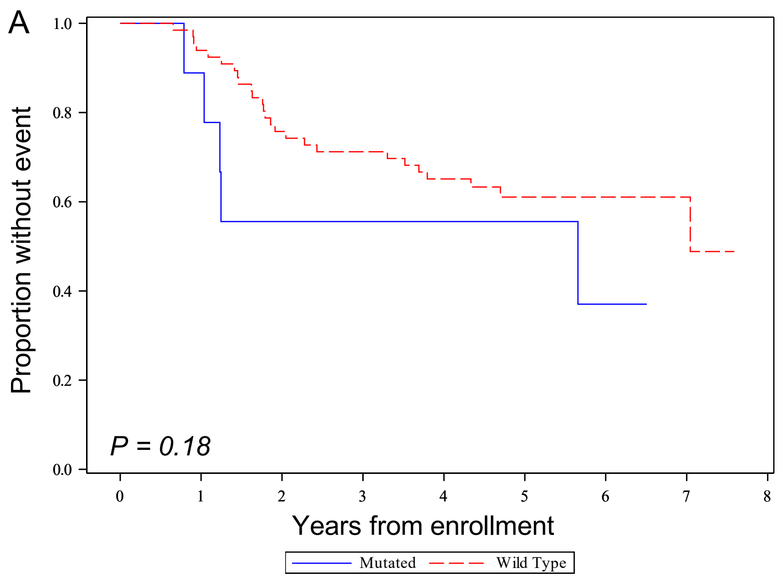

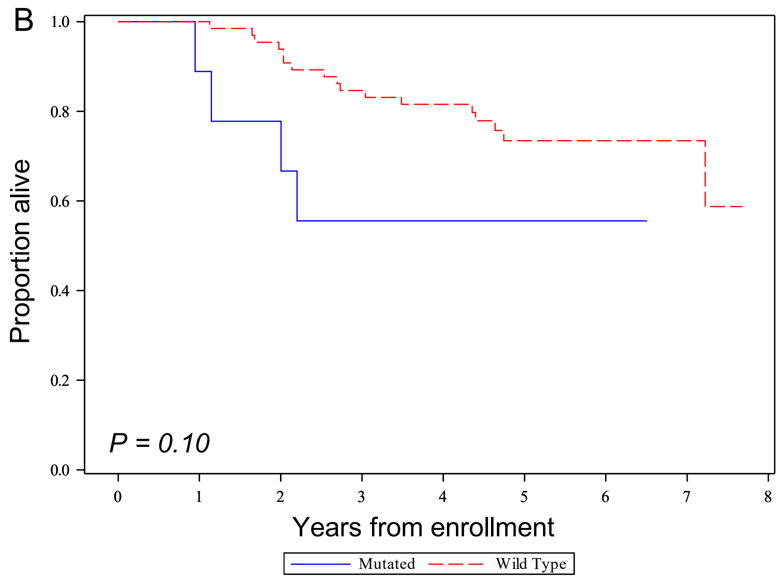

Supplemental Figure 2. Event-free survival (A) and overall survival (B) for patients with localized Ewing sarcoma stratified by *TP53* mutational status.

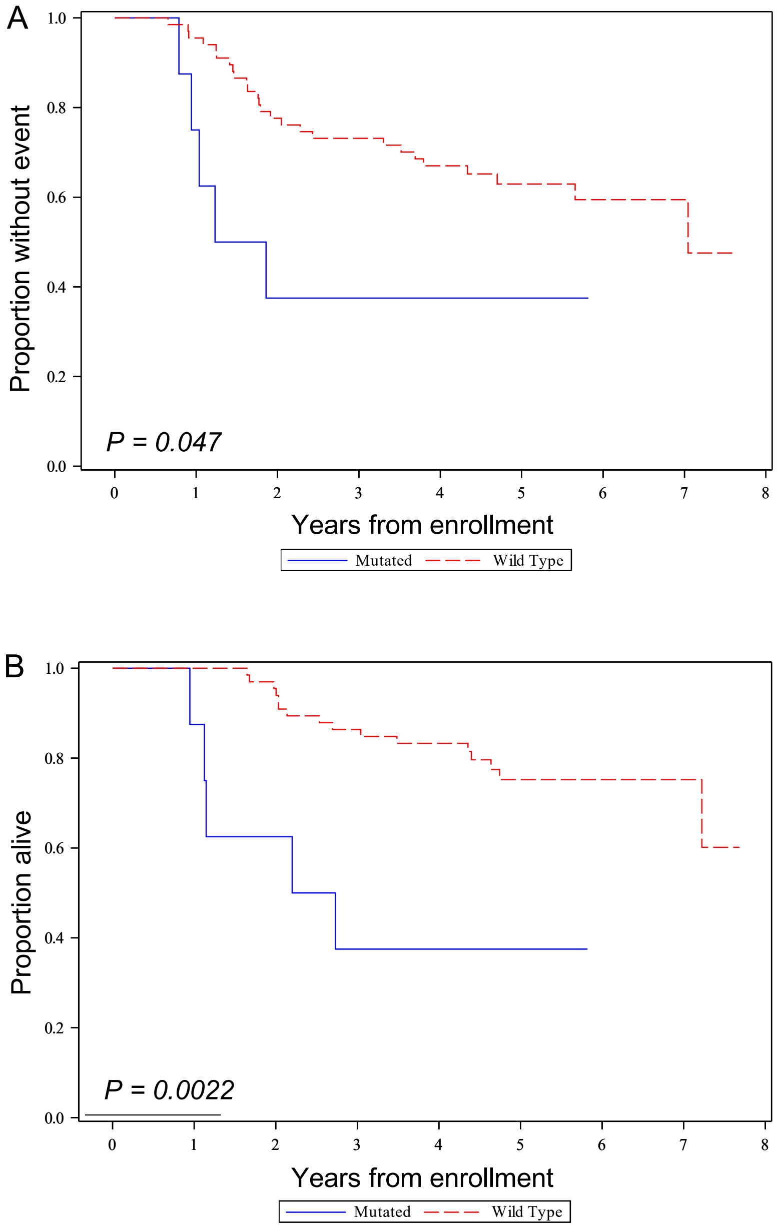

Supplemental Figure 3. EFS (A) and OS (B) stratified by *STAG2*/*TP53* mutation status groupings for patients with localized Ewing sarcoma (n=75).

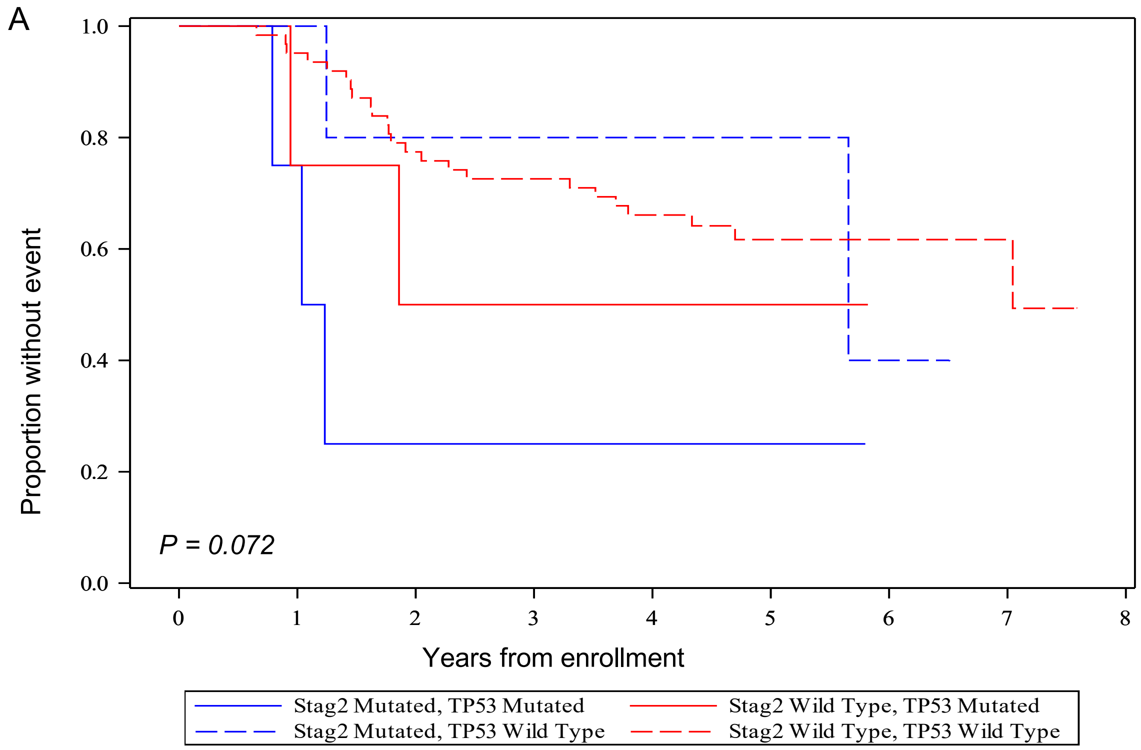

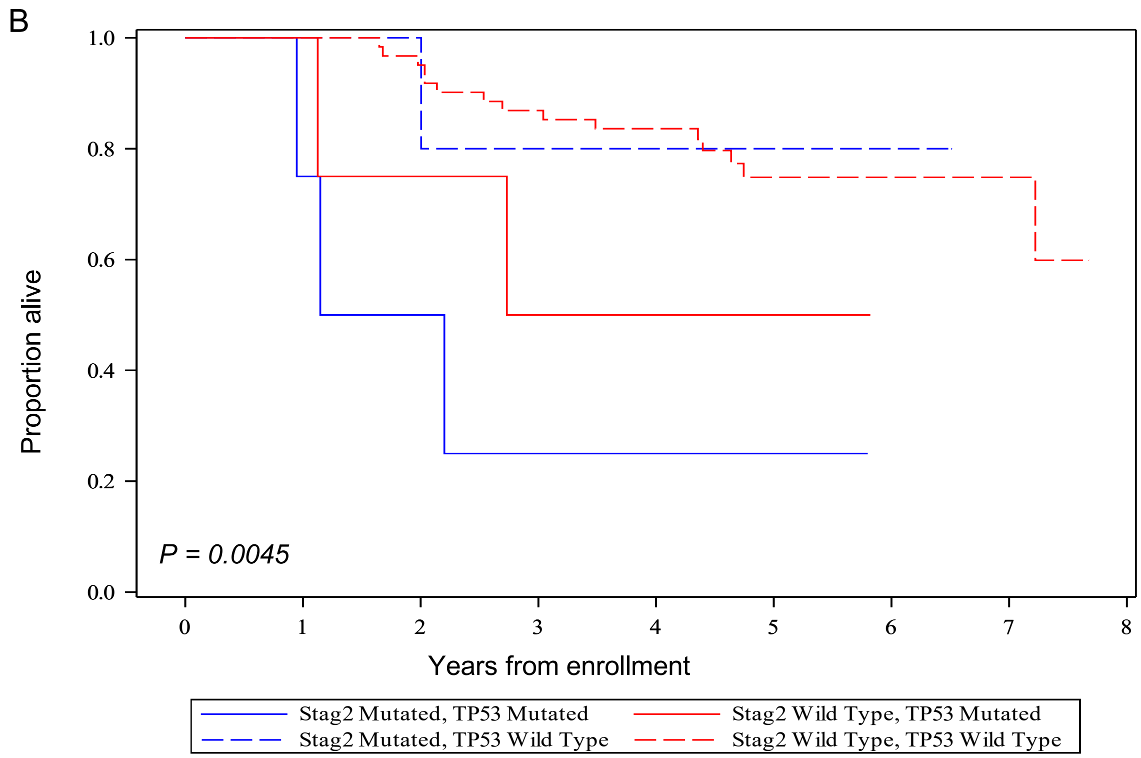

Supplement: Supplementary file 2 — Supplemental materials [file 41416_2022_1977_MOESM2_ESM.pdf]
